# Effectiveness of Omega-3 fatty acid for polycystic ovary syndrome: a systematic review and meta-analysis

**DOI:** 10.1186/s12958-018-0346-x

**Published:** 2018-03-27

**Authors:** Kailin Yang, Liuting Zeng, Tingting Bao, Jinwen Ge

**Affiliations:** 1 Hunan University of Chinese Medicine, Changsha, 410208 Hunan Province China; 2 Beijing University of Chinese Medicine, Beijing, 100029 Beijing China

**Keywords:** Omega-3 fatty acid, Polycystic ovary syndrome, Systematic review, Meta-analysis

## Abstract

**Objective:**

To assess the effectiveness and safety of omega-3 fatty acid for patients with PCOS.

**Methods:**

In this meta-analysis, data from randomized controlled trials were obtained to assess the effects of omega-3 fatty acid versus placebo or western medicine in women with PCOS. The study’s registration number is CRD42017065859. The primary outcomes included the change of homeostatic model assessment (HOMA) of insulin resistance, total cholesterol (TC), triglyceride (TG) and adiponectin.

**Result:**

Nine trials involving 591 patients were included. Comparing with the control group, omega-3 fatty acid may improve HOMA index (WMD -0.80; 95% CI -0.89, − 0.71; P<0. 00001), decrease TC and TG level [TC: (WMD -9.43; 95% CI -11.90, − 6.95; P<0. 00001); TG: (WMD -29.21; 95% CI -48.08, − 10.34; *P* = 0. 002)], and increase adiponectin level (WMD 1.34; 95% CI 0.51, 2.17; *P* = 0. 002).

**Conclusion:**

Based on current evidence, omega-3 fatty acid may be recommended for the treatment of PCOS with insulin resistance as well as high TC (especially LDL-C) and TG.

## Background

Polycystic ovary syndrome (PCOS) is a common reproductive endocrine disease estimated to affect 6–10% of women of reproductive age [1, 2], which is associated with a variety of factors, including menstrual irregularity, insulin resistance, diabetes, and obesity [3]. The pathogenesis of PCOS is not yet clear, but genetics and lifestyle factors contribute significantly to the development of the PCOS [4]. The prevalence of metabolic syndrome (Reproductive disorders, infertility, metabolic disorders) in PCOS patients is higher than that in the general population [5]. The negative effects of these PCOS-related symptoms impaired the quality of women’s life and led them to undeniable pressure.

The recommended treatments for PCOS women, especially for PCOS patients with obesity, are lifestyle and nutrition interventions and weight loss [6, 7]. The current study shows that metabolic disorders in patients with PCOS may be improved by the intervention of dietary factors such as anti-inflammatory foods [8]. Among dietary factors, omega-3 fatty acids play an important role in immune regulation, insulin sensitivity, cellular differentiation, and ovulation [8, 9]. This dietary supplement may be used for improving excessive oxidative stress-caused folliculogenesis disorder and hyperinsulinemia in women with PCOS [10–12]. Omega-3 fatty acids supplementation also has a beneficial effect on some cardiometabolic risk factors in women with PCOS [13], which is achieved through reducing the synthesis of prostaglandins by competitive inhibition of cyclooxygenase 2 (COX-2) [9] and increasing the activity of antioxidant enzymes [14, 15].

A previous systemic review and meta-analysis which reviewed the research before 2015 have evaluated the effects of omega-3 fatty acids in PCOS women, and it reported that omega-3 fatty acids may not have a beneficial effect on improving insulin resistance in women with PCOS [16]. Over time, more randomized controlled trials (RCTs) about omega-3 fatty acid were published between 2015 and 2018. However, the new RCTs [17–20] showed that omega-3 fatty acid had a beneficial effect on serum adiponectin levels, insulin resistance, serum lipid levels and so on in PCOS patients, which is contrary to the result of the previous meta-analysis [16]. Therefore, the results of systematic review and meta-analysis need to be updated. This systemic review and meta-analysis is a registered review with protocol (CRD42017065859) in PROSPERO, which aims to evaluate the effects of omega-3 fatty acid on women with PCOS.

## Methods

### Protocol

Study selection, assessment of eligibility criteria, data extraction, and statistical analysis were performed based on a predefined protocol registered on PROSPERO (CRD42017065859).

### Search strategy and selection criteria

A search strategy was designed to search all the available literature. We searched the Pubmed, ClinicalTrials, Embase, Medline Complete, Web of Science, Cochrane Library (Until Issue 12, 2017), the Chinese Science and Technology Periodical Database (VIP), the Chinese National Knowledge Infrastructure Databases (CNKI), WanFang Database (Chinese Ministry of Science & Technology), Chinese Biomedical Database (CBM), from their inception to January, 2018. The search terms included omega-3 fatty acid, ω-3 fatty acid, n-3 fatty acid, polycystic ovary syndrome, PCOS.

Studies meeting the inclusion criteria were included in this review (see Table 1).

**Table 1:** Inclusion criteria

P (Participants)	Women with a diagnosis of polycystic ovary syndrome
I (Intervention)	Omega-3 fatty acid with no limits on the type, dose, frequency and so on
C (Comparisons)	Blanks, placebo, or western medicine
O (Outcomes)	Primary: the change of homeostatic model assessment (HOMA) of insulin resistance, total cholesterol (TC), triglyceride (TG), adiponectin, adverse events
	Secondary: body mass index (BMI), fasting insulin, fasting glucose, low density lipoprotein cholesterol (LDL-C), high density lipoprotein cholesterol (HDL-C), follicle stimulating hormone (FSH), luteotropic hormone (LH), total testosterone, sex hormone-binding globulin (SHBG)
S (Study type)	Randomized controlled trials (RCTs), which assess the effects of omega-3 fatty for the treatment of PCOS (with no limits on the manner by which randomization has been achieved, on blinding or on the language of publication)

Due to the ovarian aging in post-menopausal women, studies involving post-menopausal women (over 50 years of age) were excluded, which meet the exclusion criteria.

### Data analysis

All studies were reviewed and selected independently by three reviewers (Kailin Yang, Liuting Zeng, Tingting Bao). The titles and abstracts were reviewed, and articles which did not fit the eligibility criteria were excluded. If the title or abstract appeared to meet the eligibility criteria or they could not determine its eligibility, the full texts of the articles were obtained for further evaluation. For example, the search strategy for Pubmed was present in Table 2; ten studies of twenty-one studies in Pubmed were extracted. The data were extracted independently by three reviewers (Kailin Yang, Liuting Zeng and Tingting Bao) using a standardized data extraction form. Any discrepancies between the reviewers were resolved by consensus among all four reviewers (Kailin Yang, Liuting Zeng, Tingting Bao and Jinwen Ge). The characteristics and general information were extracted and tabulated, including Authors, time of publication, intervention, comparison group, outcomes, AEs, and follow-up period.

**Table 2:** Search Strategy for Pubmed

Database	Search Strategy
Pubmed	(n-3 Fatty Acids OR n 3 Fatty Acids OR n-3 Polyunsaturated Fatty Acid OR n 3 Polyunsaturated Fatty Acid OR n-3 PUFA OR PUFA, n-3 OR n 3 PUFA OR Omega 3 Fatty Acids OR n3 PUFA OR PUFA, n3 OR n3 Polyunsaturated Fatty Acid OR n3 Oils OR n-3 Oils OR n 3 Oils OR Omega-3 Fatty Acids OR n3 Fatty Acid OR Fatty Acid, n3) AND (Ovary Syndrome, Polycystic OR Syndrome, Polycystic Ovary OR Stein-Leventhal Syndrome OR Stein Leventhal Syndrome OR Syndrome, Stein-Leventhal OR Sclerocystic Ovarian Degeneration OR Ovarian Degeneration, Sclerocystic OR Sclerocystic Ovary Syndrome OR Polycystic Ovarian Syndrome OR Ovarian Syndrome, Polycystic OR Polycystic Ovary Syndrome 1 OR Sclerocystic Ovaries OR Ovary, Sclerocystic OR Sclerocystic Ovary OR PCOS) AND (randomized controlled trial [pt] OR controlled clinical trial [pt] OR placebo [tiab] OR drug therapy [sh] OR trial [tiab] OR groups [tiab] OR clinical trials as topic [mesh: noexp] OR Clinical Trial OR random* [tiab] OR random allocation [mh] OR single-blind method [mh] OR double-blind method [mh] OR cross-over studies) NOT (animals [mh] NOT humans [mh])

If there was missing information in the paper, such as methodology, diagnosis, interventions and outcomes, reviewers would try to contact the original authors to clarify the data, or impute the missing standard deviations according to the Cochrane Handbook 5.1.0—if there were missing standard deviations, if several candidate standard deviations are available, reviewers would to use their average to impute it. (P=NS or *P* > 0.05), or reviewers imputed them by *P*-value (*P* < 0.05) [21].

The risk of bias was assessed using the risk of bias assessment tool by the Cochrane Handbook for Systematic Reviews of Interventions, version 5.1.0 [22]. The criteria consist of 7 items related to selection bias (random sequence generation and allocation concealment), performance bias (blinding of participants and personnel), detection bias (blinding of outcome assessment), attrition bias (incomplete outcome data), reporting bias (selective outcome reporting), and other sources of bias. Three reviewers (Liuting Zeng, Kailin Yang, Tingting Bao) independently performed this, and any discrepancies between the two reviewers were resolved by consensus among all four reviewers (Kailin Yang, Liuting Zeng, Tingting Bao and Jinwen Ge).

The data were analyzed using RevMan 5.3 software. The dichotomous variable measure was summarized by risk ratio (RR) with a 95% confidence interval (CI). The continuous outcomes underwent meta-analysis using mean differences (MD) and 95% CI. Heterogeneity among studies was assessed using Cochrane’s Q and I^2^ statistic [23]. When *P* > 0.1, *I*2 < 50%, we used a fixed effect model; when *P* < 0.1, I2 > 50%, we would explore the reasons for heterogeneity, perform the subgroup analysis and use a random effect model.

Primary outcomes, secondary outcomes, and adverse events (AEs) would be reported. Except menstrual cycle regulation (no research reported the outcome), all outcomes were prespecified in the study protocol.

## Results

### Results of the search

**Fig. 1:**
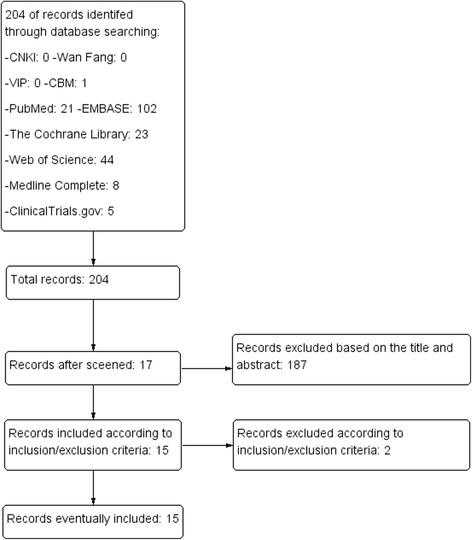
Flow diagram of searching and article selection

Our initial search identified and screened 204 articles. We excluded 187 articles based on the title and abstract and retrieved 17 articles for more detailed evaluation. From these, we excluded 2 publications and included 15 studies in our review (Fig. 1).

### Description of included trials and risk of Bias in included studies

Nine RCTs with 591 participants met the inclusion criteria. There are four records whose data [7, 12, 24, 25] derived from the same clinical trial, so we counted them as one RCT (Mohammadi 2012 [25]). All of them were parallel-group RCTs. Because there are three groups in Karakas’s research [17], two of them are trial groups, while one is control group; according to the Cochrane Handbook 5.1.0, we split the shared control group into two groups with smaller sample size [21], and include the two reasonably independent comparisons (Karakas 2016 a and Karakas 2016 b). Study characteristics are presented in Table 3.

**Table 3:** The characteristics of the included studies

Study	Sample size		Intervention		Relevant outcomes	Mean age (years)		Mean BMI (baseline)		Duration
	Trial group	Control group	Trial group	Control group		Trial group	Control group	Trial group	Control group	
Mohammadi 2012 [25]	30	31	Omega-3 fatty acids 4000 mg	Paraffin oil (placebo) 2000 mg	BMI, TG, TC, LDL-C, HDL-C, Adiponectin, fasting insulin, fasting glucose, the change of HOMA	27.33 ± 4.27	27.73 ± 4.53	28.67 ± 3.21	28.77 ± 2.92	8 weeks
Karakas 2016 [17]	34	17	Omega-3 fatty acids (including fish oils and flaxseed oils)	Soybean oil (placebo)	BMI, TG, TC, LDL-C, HDL-C, fasting insulin, fasting glucose, the change of HOMA	Fish oils: 31.7 ± 7.8; Flaxseed oils: 29.4 ± 6.6	28.9 ± 4.1	Fish oils: 36.3 ± 7.8; Flaxseed oils: 35.0 ± 10.3	33.2 ± 7.4	6 weeks
Nadjarzadeh 2015 [18]	39	39	Omega-3 fatty acids 900 mg	Paraffin oil (placebo) 3000 mg	BMI, Adiponectin, FSH, LH	26.9 ± 5.9	26.9 ± 5.0	31.46 ± 5.74	31.88 ± 3.86	12 weeks
Nadjarzadeh 2013 [26]	39	39	Omega-3 fatty acids 900 mg	Paraffin oil (placebo) 3000 mg	Total testosterone, SHGB	26.9 ± 5.9	26.9 ± 5.0	31.46 ± 5.74	31.88 ± 3.86	12 weeks
Rahmani 2017 [19]	34	34	Omega-3 fatty acids 1000 mg + Vitamin E 400 IU + Metformin	Placebos + Metformin	BMI, TG, TC, LDL-C, HDL-C, FSH, LH	24.9 ± 5.5	26.6 ± 5.6	28.4 ± 4.4	29.0 ± 6.5	12 weeks
Ebrahimi 2017 [20]	34	34	Omega-3 fatty acids 1000 mg + vitamin E 400 IU	Placebos	BMI, SHGB, fasting insulin, fasting glucose, the change of HOMA, total testosterone	23.8 ± 4.6	25.2 ± 5.2	28.0 ± 4.3	28.5 ± 6.6	12 weeks
Khani 2017 [8]	43	44	Omega-3 fatty acids 2000 mg	Olive oil (placebo) 2000 mg	BMI, TG, TC, LDL-C, HDL-C, fasting glucose	31.04 ± 5.04	29.23 ± 6.73	31.8 ± 3.61	31.79 ± 3.6	24 weeks
Mirmasoumi 2017 [27]	30	30	Omega-3 fatty acids 2000 mg + Metformin 500 mg	Paraffin oil (placebo) 1000 mg + Metformin 500 mg	BMI, TG, TC, LDL-C, HDL-C, fasting insulin, fasting glucose, the change of HOMA, total testosterone, SHBG, adverse events	28.4 ± 6.4	27.0 ± 3.2	26.9 ± 5.1	26.7 ± 5.3	12 weeks
Jamilian 2018 [28]	20	20	Omega-3 fatty acids 1000 mg + Vitamin E 400 IU	Paraffin oil (placebo)	BMI, the change of HOMA	22.3 ± 4.7	24.4 ± 4.7	28.8 ± 5.1	26.5 ± 5.9	12 weeks

**Fig. 2:**
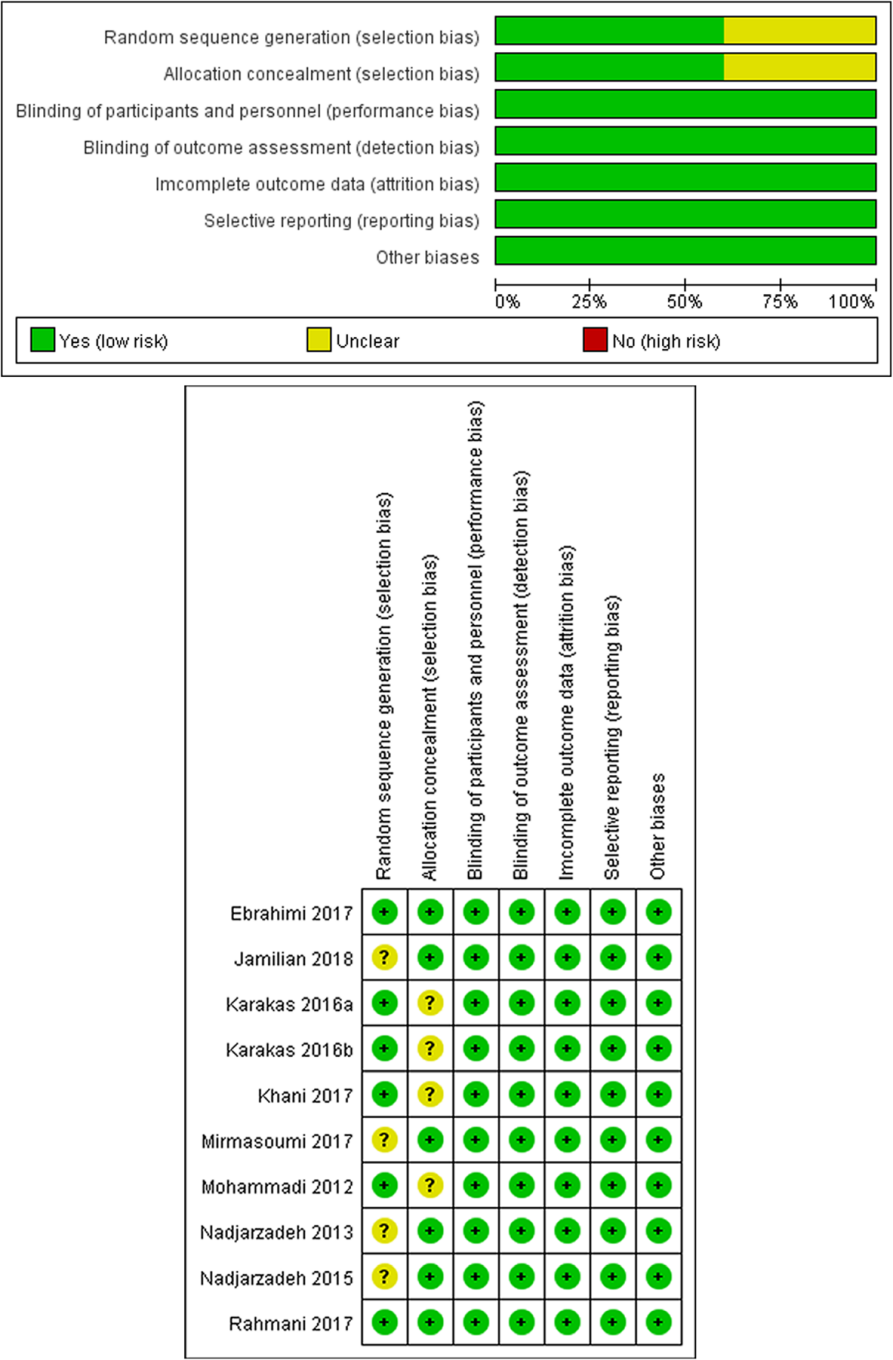
The risk of bias

Among the 9 included RCTs, four studies [18, 26–28] adopted unclear randomization procedures, while the others described adequate methods of random sequence generation: the block randomization procedure [25], website [17], random-maker software “random allocation” [8] or computer-generated randomization list [19, 20]; we rated the five studies as having an unclear risk of bias, while the others trials were at low risk of bias. We rated three trials [8, 17, 25] as having an unclear risk of bias because they did not describe an acceptable method of allocation concealment; because of that the others [19, 20, 26–28] described that drugs in trial groups and control groups were similar in shape, size and so on that the patients and researcher were not aware until the end of the analysis, we rated them as having a low risk of bias. For participant and outcome assessment blinding, five trials were unclear [17–20, 26], but they used objective measures (e.g. TG, TC, adiponectin) and the outcome is not likely to be influenced by the lack of blinding, while the rest one studies used blinding; thus, we gave a low risk of bias for all. None of trials missed data and incompletely reported the outcomes, therefore we gave a low risk of bias. Other sources of bias were at low risk in all of the included studies. A graphical summary of the risks of bias assessment is presented in Fig. 2.

### Primary outcomes

**Fig. 3:**
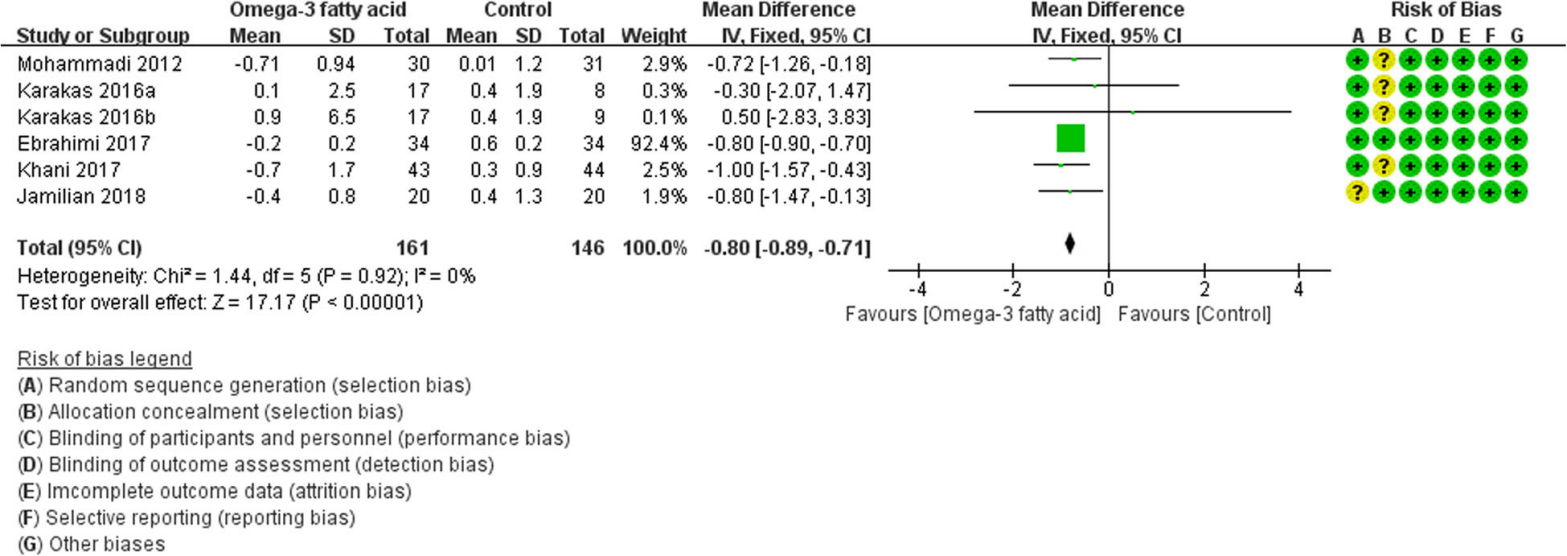
The Change of Homeostasis Model Assessment of Insulin Resistance

Five RCTs [8, 17, 20, 25, 28] reported the change of HOMA at the end of treatment. Due to the low heterogeneity, we used fix effect model. In this index, it can be found that in improving insulin resistance, omega-3 fatty acid is better [the change of HOMA: (WMD -0.80; 95% CI -0.89, − 0.71; *P*<0. 00001)] (Fig. 3).

**Fig. 4:**
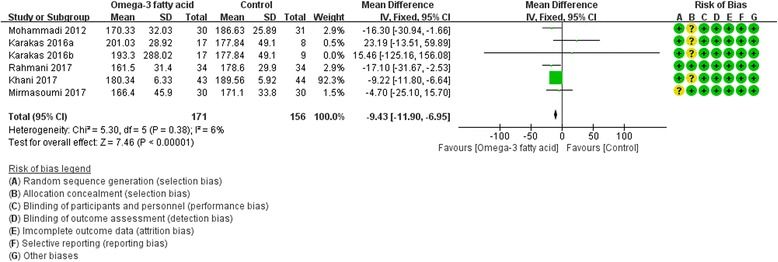
Total Cholesterol

Five RCTs [8, 17, 19, 25, 27] reported total cholesterol. We used fix effect model. According to the result, compared with the control group, omega-3 fatty acid is better in decrease TC [TC: (WMD -9.43; 95% CI -11.90, − 6.95; *P*<0. 00001)] (Fig. 4).

**Fig. 5:**
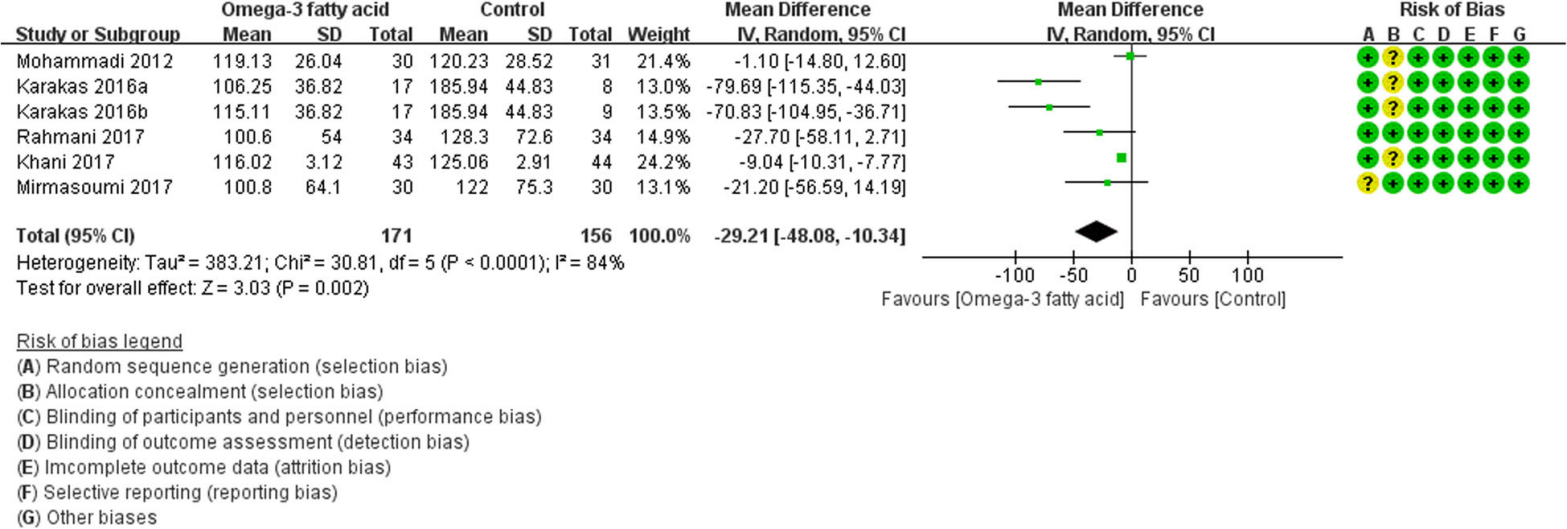
Triglyceride

Five RCTs [8, 17, 19, 25, 27] reported triglyceride (TG) level. Due to the heterogeneity (Tau^2^ = 383.21, I^2^ = 84%, *P*<0.0001), we used random effect model. It seems like that compared with the control group, omega-3 fatty acid can decrease the TG level in PCOS patients [TG: (WMD -29.21; 95% CI -48.08, − 10.34; *P* = 0. 002)] (Fig. 5).

**Fig. 6:**
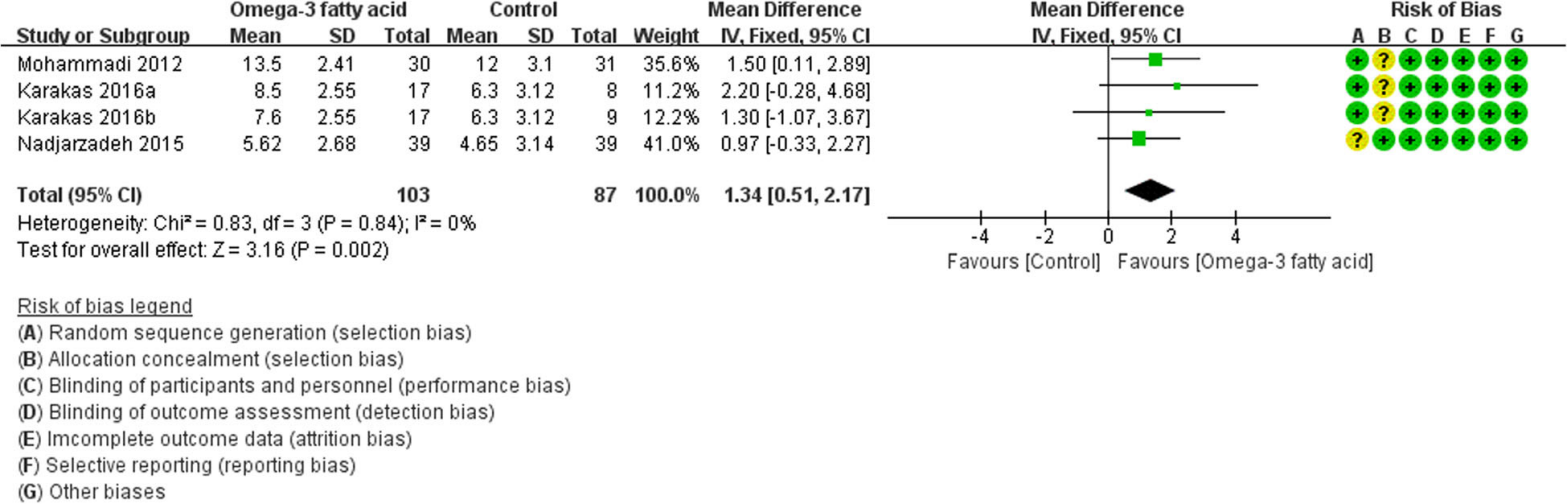
Adiponectin

Three RCTs [17, 18, 25] reported adiponectin level. We used fix effect model. According to the results, compared with the control group, omega-3 fatty acid can increase the adiponectin level in PCOS patients [Adiponectin: (WMD 1.34; 95% CI 0.51, 2.17; *P* = 0. 002)] (Fig. 6).

### Secondary outcomes

**Fig. 7:**
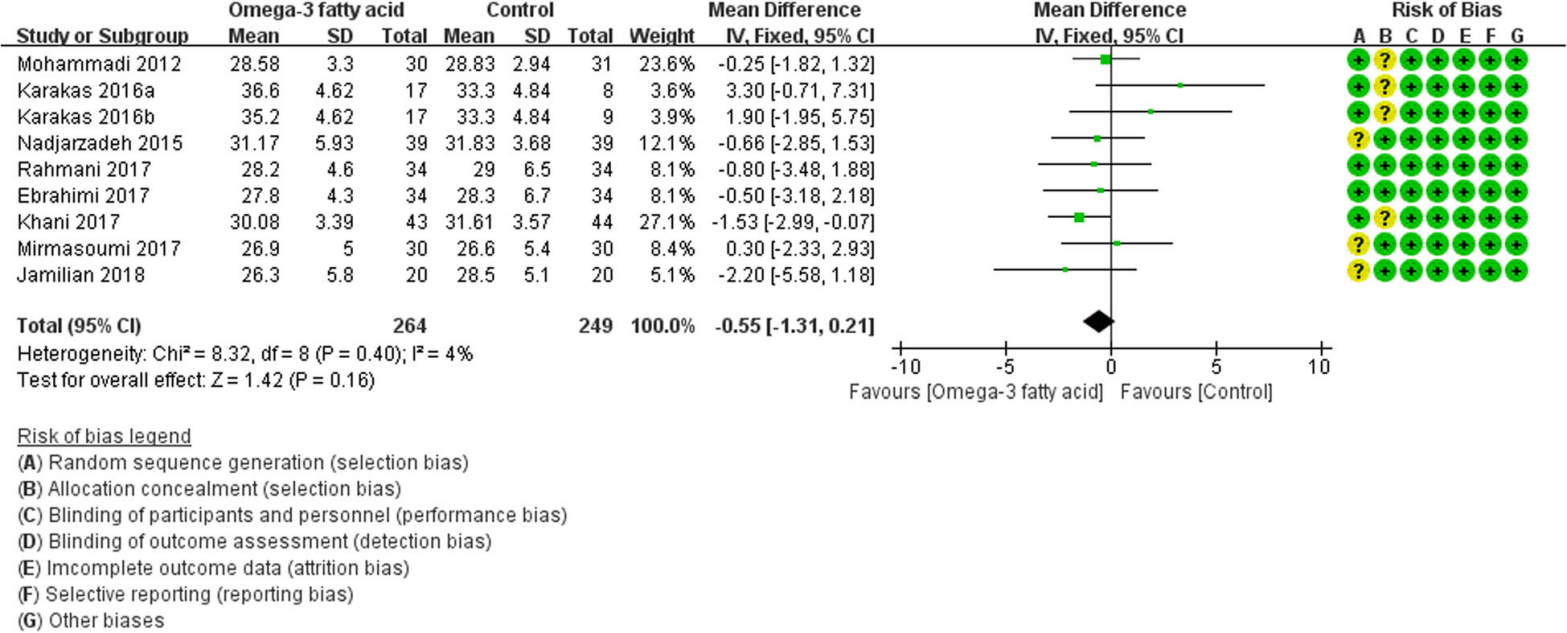
BMI

Eight RCTs [8, 17–20, 25, 27, 28] reported BMI. We used fix effect model. In this index, there is not strong evidence that the omega-3 fatty acid has an effect on BMI because there was no statistical difference [BMI: (WMD -0.55; 95% CI -1.31, 0.21; *P* = 0. 16)] (Fig. 7).

**Fig. 8:**
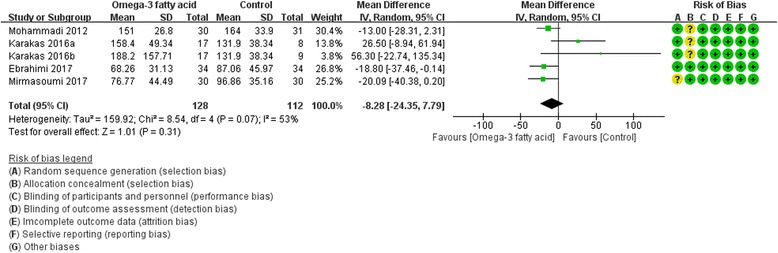
Fasting insulin

**Fig. 9:**
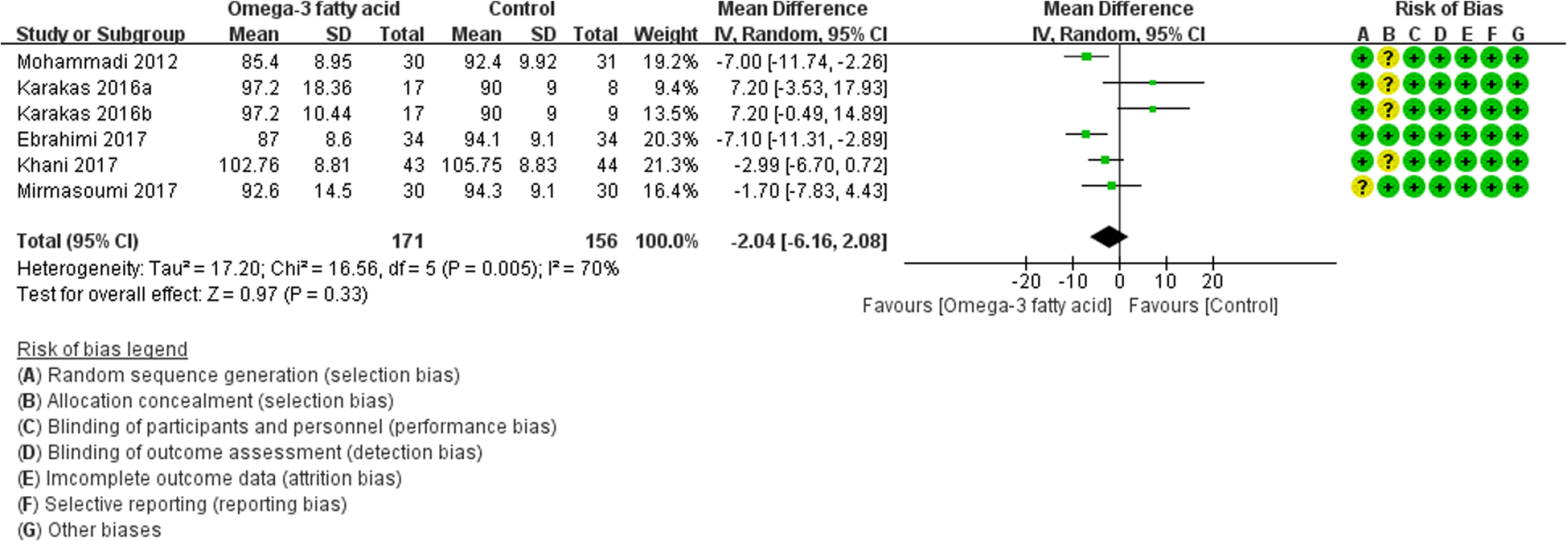
Fasting glucose

Four RCTs [17, 19, 20, 27] reported fasting insulin and five RCTs reported fasting glucose [8, 17, 19, 20, 27] at the end of treatment. Due to the heterogeneity [Fasting insulin: (Tau^2^ = 159.92, I^2^ = 53%, *P* = 0.07); Fasting glucose: (Tau^2^ = 17.20, I^2^ = 70%, *P* = 0.005)], we used random effect model. For fasting insulin, compared with the control group, there is not strong evidence that the omega-3 fatty acid has an effect on hyperinsulinemia because there was no statistical difference (WMD -8.28; 95% CI -24.35, 7.79; *P* = 0. 31) (Fig. 8). And for fasting glucose, there is also not strong evidence that the omega-3 fatty acid has an effect on fasting glucose because there was no statistical difference (WMD -2.04; 95% CI -6.16, 2.08; *P* = 0. 33) (Fig. 9).

**Fig. 10:**
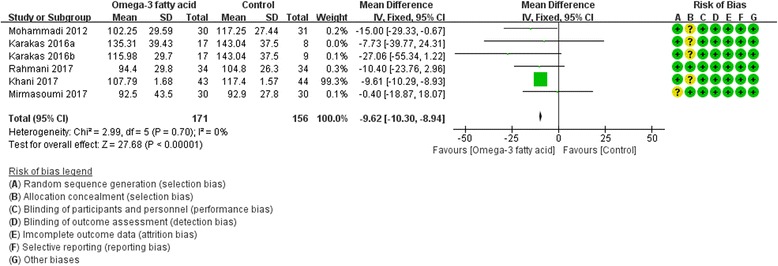
LDL-C

**Fig. 11:**
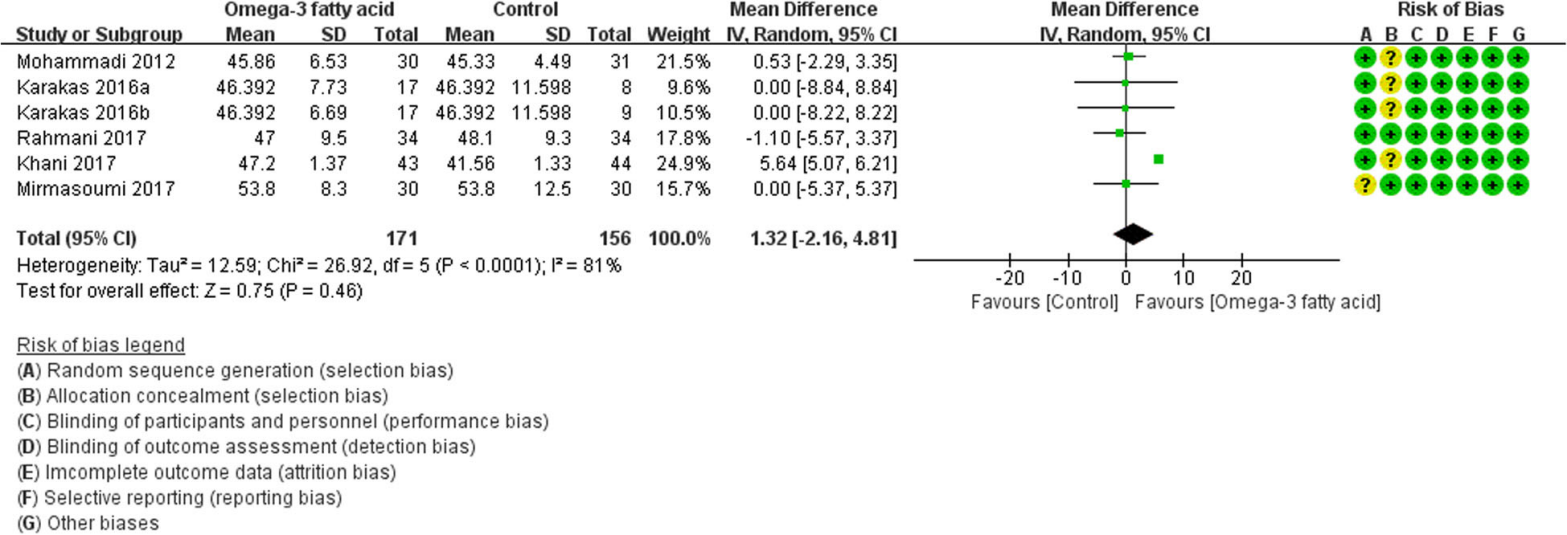
HDL-C

Five RCTs [8, 17, 19, 25, 27] reported LDL-C and HDL-C at the end of treatment. For LDL-C, we used fix effect model, and compared with the control group, the omega-3 is likely to decrease LDL-C (WMD -9.62; 95% CI  -10.30,  -8.94; *P* <0.00001) (Fig. 10). However, for HDL-C, we used random effect model because of its high heterogeneity (Tau^2^ = 12.59, I^2^ = 81%, P<0.0001), and there is also not strong evidence that the omega-3 fatty acid has an effect on fasting glucose because there was no statistical difference (WMD 1.32; 95% CI -2.16, 4.81; *P* = 0. 46) (Fig. 11).

**Fig. 12:**
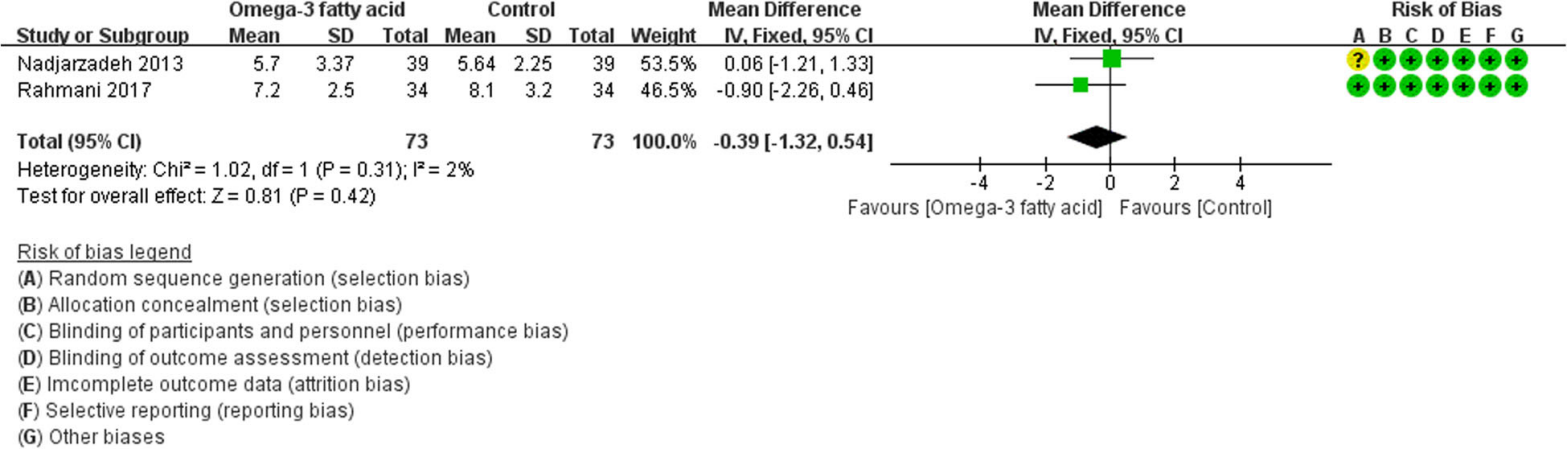
FSH

**Fig. 13:**
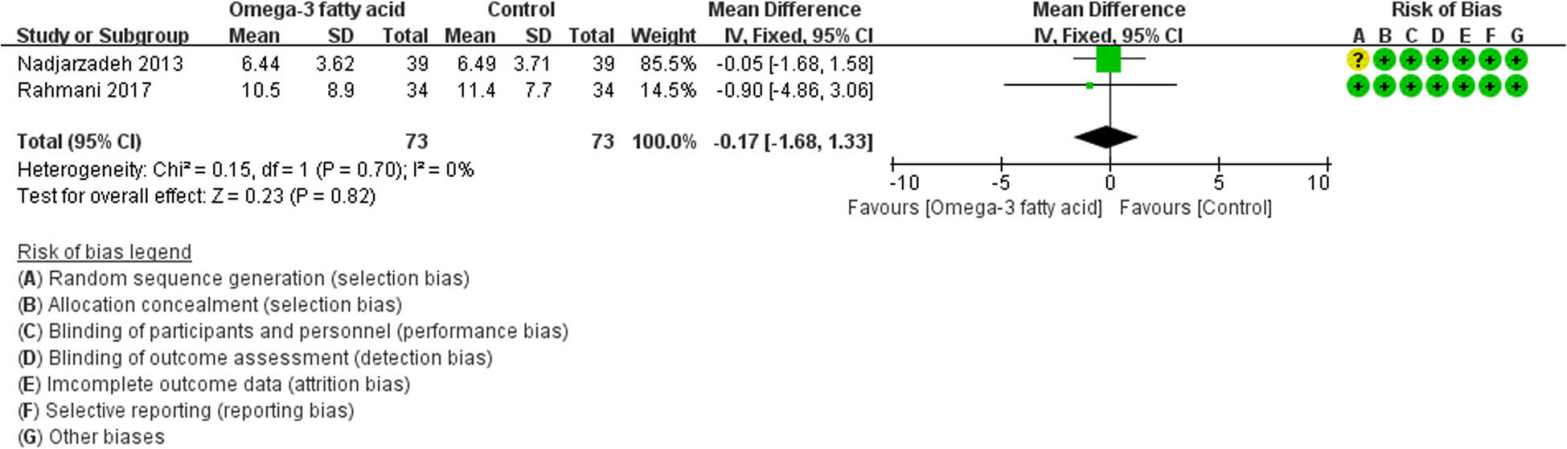
LH

**Fig. 14:**
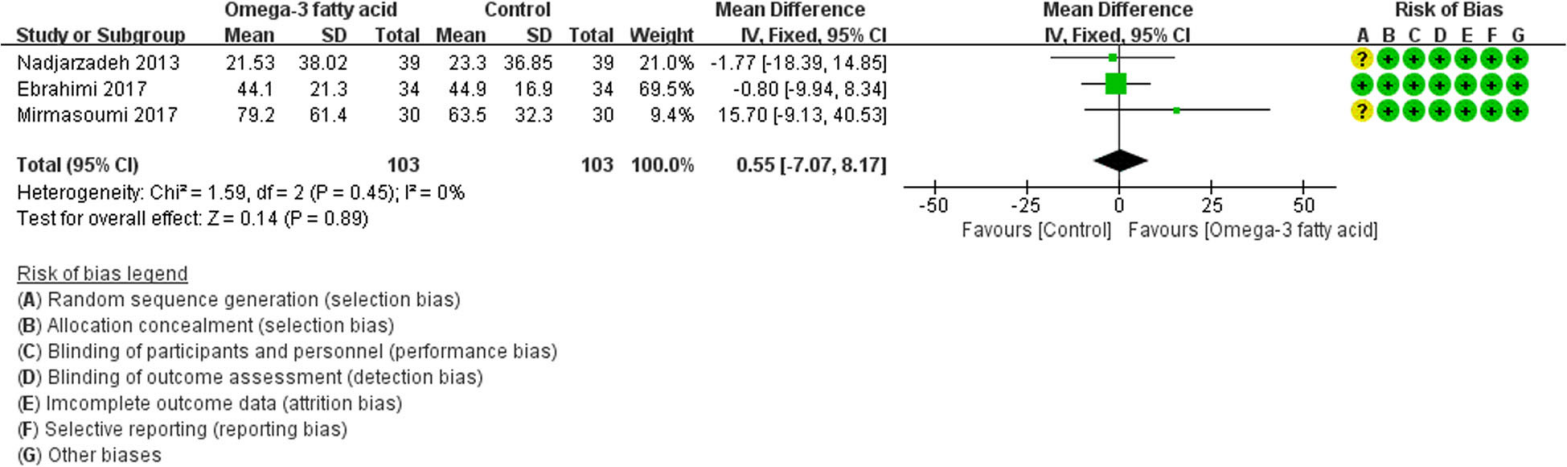
SHGB

**Fig. 15:**
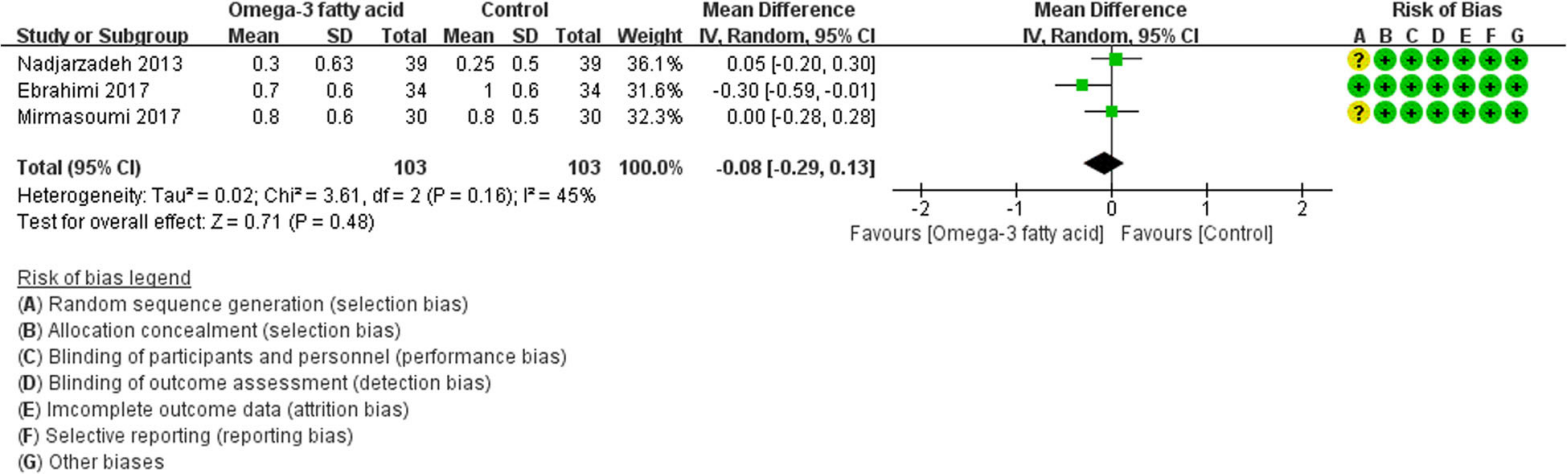
Total testosterone

Only two RCTs [19, 26] reported FSH and LH, and three RCTs [19, 20, 27] reported SHGB and total testosterone. For FSH, LH and SHBG, we used fix effect model; while for total testosterone, due to the heterogeneity (Tau^2^ = 0.02, I^2^ = 45%, *P* = 0.16), we used random effect model. However, for all of these indexes, there is also not strong evidence that the omega-3 fatty acid has an effect on fasting glucose because there was no statistical difference. [FSH: (WMD -0.39; 95% CI -1.32, 0.54; P = 0. 42); LH: (WMD -0.17; 95% CI -1.68, 1.33; *P* = 0. 82); SHGB: (WMD 0.55; 95% CI -7.07, 8.17; *P* = 0. 89); total testosterone: (WMD -0.08; 95% CI -0.29, 0.13; *P* = 0.49)] (Figs. 12, 13, 14 and 15).

### Adverse events

Only one study [27] reported AEs and the rest of them did not mention AEs at all. And this study mentioned that there were no serious AEs reported.

## Discussions

This systematic review and meta-analysis including 9 RCTs analyzes the effectiveness of omega-3 fatty acid for PCOS. Compared with the control group, omega-3 fatty acid may improve insulin resistance (improve HOMA index and increase adiponectin level), and decrease TC, TG, LDL-C. Meanwhile, there is not strong evidence that the omega-3 fatty acid has an effect on BMI, fasting insulin, fasting glucose, HDL-C, FSH, LH, SHGB and total testosterone. As PCOS is closely associated with insulin resistance and hyperandrogenism [29–31], based on current evidence, omega-3 fatty acid may be recommended for the treatment of PCOS with insulin resistance or/and high TC (especially LDL-C) and TG. While this finding seems promising, it should be interpreted with caution mainly due to the unclear risk of bias for selection bias (random sequence generation and allocation concealment) and a small number of participants. Although comparing with control group, there is not strong evidence that the omega-3 fatty acid has an effect on BMI, fasting insulin, fasting glucose, HDL-C, FSH, LH, SHGB and total testosterone, it does not mean there is no medical significance. Instead, it may mean that omega-3 fatty acid may be the safer or cheaper treatment options.

Only one study [27] reported AEs and the rest of them did not mention AEs at all. This RCT reports that no relevant side effect was recorded during the therapy. However, the absence of information on AEs does not mean that the intervention is safe [32]. Thus, although based on current evidences, we consider that omega-3 fatty acid is a relatively safe treatment, we cannot assure it. Future clinical trials are required to report AEs with more explanations [33].

PCOS is one of the most common endocrine disorders that women suffer from [34], which is closely related to insulin resistance and hyperandrogenism [4–6]. The relationship between insulin resistance and hyperandrogenism is that insulin resistance can stimulate the production and secretion of androgens and ovarian failure [35–37]. Therefore, improving insulin resistance is considered to be of quit importance for PCOS [35]. Omega-3 fatty acids are the very substance that increases the sensitivity to insulin by producing and secreting anti-inflammatory adipokine (such as adiponectin) and reducing inflammation and proinflammatory cytokines [25, 28–39], which has been revealed in our meta-analysis. Omega-3 fatty acids can also reduce cholesterol absorption and LDL-C synthesis, improve LDL receptor activity in liver, and increase fractional rate of catabolism of LDL-C [40, 41]. Therefore, omega-3 fatty acid supplementation had a beneficial effect on some cardiometabolic risk factors in women with PCOS [13].

Comparing with previous reviews [16], the strengths of this systematic review and meta-analysis are that it indicates that omega-3 fatty acid may be suitable for the treatments of PCOS with insulin resistance or/and high TC (especially LDL-C) and TG. And this review included six recent (2016–2018) RCTs [8, 17, 19, 20, 27, 28]. A study concluded that publication bias is smaller in meta-analysis of more recent studies [42]; therefore, the risk of publication bias of this review may not be high. The limitations include the small number of trials, the small number of participants and the high heterogeneity for some outcomes (such as TG and HDL-C). The high level of heterogeneity was explored using sensitivity analysis for risk of bias, however, upon removal of the RCTs with medium risk of bias, there was no difference in the direction of effect or the heterogeneity. The heterogeneity may come from placebo effects or other places. Meanwhile, study duration is generally short-to-medium term (mostly 12 weeks), the long-term efficacy of omega-3 fatty acid is temporarily uncertain. Additionally, due to none of trials that reported AEs, the safety of omega-3 fatty acid should be interpreted with caution. Finally, the absolute treatment effects should also be interpreted with caution because the number of participants is small and it may not be generalizable to all types of PCOS. Further rigorously designed studies are needed to confirm the effectiveness and safety of omega-3 fatty acid in patients with PCOS. Furthermore, the individual patient data (IPD) meta-analysis, which would allow analysis to control for all types of heterogeneity due to differences in studies and/or patients and may be potentially more reliable than aggregate data meta-analysis, are also needed in the future [43, 44].

## Conclusion

Our systematic review and meta-analysis provides evidence that omega-3 fatty acid may be a novel drug for PCOS patients. And based on current evidence, omega-3 fatty acid may be recommended for the treatment of PCOS with insulin resistance as well as high TC (especially LDL-C) and TG. However, current RCTs have limitations, including small sample sizes and short duration. The benefits from long term treatment of omega-3 fatty beyond 6 months remain to be defined by future studies. Meanwhile, more randomized, double-blind, large sample size trials of omega-3 fatty for PCOS are needed in the future to confirm or modify the result of this work.
